# H3K4me3 changes occur in cell wall genes during the development of *Fagopyrum tataricum* morphogenic and non-morphogenic calli

**DOI:** 10.3389/fpls.2024.1465514

**Published:** 2024-09-25

**Authors:** Alicja Tomasiak, Artur Piński, Anna Milewska-Hendel, Ignasi Andreu Godall, Natalia Borowska-Żuchowska, Joanna Morończyk, Jordi Moreno-Romero, Alexander Betekhtin

**Affiliations:** ^1^ Institute of Biology, Biotechnology and Environmental Protection, Faculty of Natural Sciences, University of Silesia in Katowice, Katowice, Poland; ^2^ Centre for Research in Agricultural Genomics (CRAG), CSIC-IRTA-UAB-UB, Barcelona, Spain; ^3^ Departament de Bioquímica i Biologia Molecular, Universitat Autònoma de Barcelona, Cerdanyola del Vallès, Barcelona, Spain

**Keywords:** callus, cell wall, ChIP-sequencing, *Fagopyrum tataricum*, histone modification, morphogenic, native chromatin immunoprecipitation, non-morphogenic

## Abstract

Epigenetic changes accompany the dynamic changes in the cell wall composition during the development of callus cells. H3K4me3 is responsible for active gene expression and reaction to environmental cues. Chromatin immunoprecipitation (ChIP) is a powerful technique for studying the interplay between epigenetic modifications and the DNA regions of interest. In combination with sequencing, it can provide the genome-wide enrichment of the specific epigenetic mark, providing vital information on its involvement in the plethora of cellular processes. Here, we describe the genome-wide distribution of H3K4me3 in morphogenic and non-morphogenic callus of *Fagopyrum tataricum*. Levels of H3K4me3 were higher around the transcription start site, in agreement with the role of this mark in transcriptional activation. The global levels of methylation were higher in the non-morphogenic callus, which indicated increased gene activation compared to the morphogenic callus. We also employed ChIP to analyse the changes in the enrichment of this epigenetic mark on the cell wall-related genes in both calli types during the course of the passage. Enrichment of H3K4me3 on cell wall genes was specific for callus type, suggesting that the role of this mark in cell-wall remodelling is complex and involved in many processes related to dedifferentiation and redifferentiation. This intricacy of the cell wall composition was supported by the immunohistochemical analysis of the cell wall epitopes’ distribution of pectins and extensins. Together, these data give a novel insight into the involvement of H3K4me3 in the regeneration processes in *F. tataricum in vitro* callus tissue culture.

## 1 Introduction

Plant tissue culture demonstrates remarkable capacity for cellular dedifferentiation and subsequent regeneration (Lee and Seo, 2018). Callus is a highly proliferative cell mass that is often considered dedifferentiated. Its growth and development under *in vitro* conditions depend on the activity of meristematic cells, which are capable of undergoing dedifferentiation and redifferentiation under specific conditions (Feher, 2019; Long et al., 2022). Callus tissue undergoes genomic reprogramming during its development, transitioning to a state allowing for the embryogenic cell formation and pluripotency (Ikeuchi et al., 2013). Consequently, the callus can exhibit the ability for direct organogenesis and embryogenesis. For those reasons, callus tissue grown *in vitro* offers a valuable model for exploring plant meristematic differentiation due to its sustained cell division, expansion and growth capacity (Feher, 2019). Cell fate transitions are governed by alterations of epigenetic patterns (Birnbaum and Roudier, 2017; Lee and Seo, 2018; Feher, 2019). Among other mechanisms, histone post-translational modifications are pivotal in governing gene transcription throughout the phases of cell growth and development, as well as in orchestrating the cell’s reaction to both external and internal stimuli (Desvoyes et al., 2014; Birnbaum and Roudier, 2017; Bednarek and Orłowska, 2019). The trimethylation of histone H3 at lysine 4 (H3K4me3) mark in plants regulates gene expression and transcriptional memory, influences numerous developmental processes as well as stress responses (Foroozani et al., 2021). Multiple *Arabidopsis* mutants exhibit a locus-specific absence of H3K4me3, which manifests diverse developmental irregularities. These observations indicated that H3K4me3 likely plays crucial roles in upholding the typical expression of numerous genes (Guo et al., 2010). Accordingly, it has been shown an enrichment of H3K4me3 on multiple genes during the *Arabidopsis* leaf-to-callus transition (Hong et al., 2024). Previous research conducted by our group revealed that the levels of H3K4me3 mark in the morphogenic callus of *F. tataricum* increased with the acquisition of the regeneration potential (Tomasiak et al., 2023).

Among different changes, cellular reprogramming processes involve reorganising the cell wall components and specific gene expression (El-Tantawy et al., 2013; Su and Higashiyama, 2018). The primary cell wall is a highly dynamic structure and plays a crucial role in the life cycle of a plant (Sala et al., 2019; Leszczuk et al., 2023). It provides mechanical support to cells, participates in their growth and differentiation, and controls morphogenesis and the whole plant’s architecture (Jamet et al., 2006; Corral-Martinez et al., 2016). The primary cell wall is structurally heterogeneous. The main components are pectins and glycoproteins such as hydroxyproline-rich glycoproteins (HRGPs) that include arabinogalactan proteins (AGPs) and extensins (Keller, 1993). By influencing the physical properties of the cell wall, they contribute to processes such as cell adhesion, expansion, intercellular communication and regulatory functions (Smertenko and Bozhkov, 2014). Extensins are one of the most crucial wall glycoproteins, which significantly impact growth processes and are essential for primary cell wall structure and plant development. They form networks that influence the extensibility of the cell wall, and their increased content in the cell wall causes the termination of cell growth (Sala et al., 2019). Our previous research demonstrated the presence of extensins on the surface of morphogenic and non-morphogenic calli of *F. tataricum*, suggesting that they are crucial for cell proliferation, differentiation, cell extension (Betekhtin et al., 2019). Diverse groups of enzymes are also involved in the cell wall reorganisation and metabolism. Pectin methyl esterase inhibitors (PMEIs) are proteins that inhibit pectin methylesterase (PME) enzymes. By inhibiting their activity, PMEI maintains pectin in a more methylated state, affecting the rigidity and porosity of the cell wall (Wormit and Usadel, 2018). Moreover, PMEs may affect the pH and consequently activate other cell wall degrading enzymes, thereby facilitating cell expansion, growth, and separation (Betekhtin et al., 2018). Overexpression of *PMEI* led to the development of callus-like structures without the addition of exogenous auxin, indicating that this cluster participates in cell reprogramming during callus formation (Xu et al., 2018). Polygalacturonases play a vital role in pectin degradation, essential for cell wall loosening and remodelling during growth and development (Shin et al., 2021). Studies on poplar established that overexpression of genes associated with cell wall degradation and loosening was upregulated during callus formation, which led to reduced pectin content. Researchers concluded that cell wall degradation and biosynthesis were required for cell fate transition (Cao et al., 2021; Zhang et al., 2024). Peroxidases take part in the formation and cross-linking of lignin and other components of the cell wall, providing structural support and rigidity. They regulate cell elongation, differentiation, and development (Nicolas et al., 2003). A study on *Linum usitatissimum* showed that fluctuations in peroxidase levels accompany shoot formation in callus. An increase in peroxidase activity was noticed during the induction of shoots callus, indicating a role for peroxidases in differentiation processes (McDougall et al., 1992). Altogether, substantial evidence was demonstrated that the dynamic changes in the cell wall composition occur during dedifferentiation and redifferentiation.

In this work, we aimed to link the changes of histone modifications in callus with cell wall related genes. To address that, we used chromatin immunoprecipitation (ChIP), a powerful technique to study the interactions of nuclear proteins with DNA (Gade and Kalvakolanu, 2012). The interplay of proteins and DNA is fundamental to numerous cellular processes, including DNA replication, repair, genomic stability maintenance, mitotic chromosome segregation, and gene expression regulation (Collas, 2010). ChIP-sequencing (ChIP-seq) is employed to identify the presence of chromatin-related proteins (e.g. histone modifications, transcription factors) along the genome (Nakato and Sakata, 2021). Here, we used the ChIP-seq for two types of *F. tataricum* calli, morphogenic (MC) and non-morphogenic (NC) to assess the enrichment of H3K4me3 genome-wide, as well as study specific loci of the genes coding for the cell wall proteins, to follow the dynamics of this histone mark in both calli during the transition from non-embryogenic to an embryogenic state. We also performed the immunohistochemical staining to compare the chemistry of cell wall components between calli developmental stages. Contrasting the epigenetic profiles of callus with different capacity for morphogenesis unveils shifts in chromatin state and the impact of diverse epigenetic changes on growth and development. This comparison enables the identification of pivotal cell wall genes associated with plant growth and development, offering fresh perspectives on how these processes are regulated.

## 2 Materials and methods

### 2.1 Plant material

The seeds of *F. tataricum*, sample k-17 were acquired from the collection of the N.I. Vavilov Institute of Plant Genetic Resources, Saint Petersburg, Russia. *F. tataricum* MC was induced from immature zygotic embryos (**Supplementary Figure S1A**). NC of *F. tataricum* appears on the surface of MC after approximately two years of culture (**Supplementary Figure S1B**) (Betekhtin et al., 2017). Calli were cultivated in the dark in an incubator at 25°C ± 1 on an RX medium. It composed of Gamborg B5, including vitamins (Duchefa, Netherlands) (Gamborg et al., 1968), 2 g L^−1^ N-Z amine A (Sigma-Aldrich, USA), 2 mg L^−1^ 2,4-dichlorophenoxyacetic acid (2,4-D, Sigma-Aldrich, USA), 0.2 mg L^−1^ kinetin (KIN, Sigma-Aldrich, USA), 0.5 mg L^−1^ 3-indoleacetic acid (IAA, Sigma-Aldrich, USA), 0.5 mg L^−1^ 1-naphthaleneacetic acid (NAA, Sigma-Aldrich, USA), 25 g L^−1^ sucrose (Chempur, Poland) and 7 g L^−1^ phyto agar (Duchefa, Netherlands). MC and NC were subcultured every four and two weeks, respectively.

### 2.2 Cross-linked chromatin immunoprecipitation and bioinformatic analysis

#### 2.2.1 Tissue preparation

MC and NC were harvested on eleventh day of cultivation and fixed according to the Diagenode s. a. (Belgium) company guidelines. Briefly, the tissues were rinsed in ddH_2_O, dried with paper towels, and placed in a crosslinking bag, which was then put in a 50 ml falcon tube with the crosslinking buffer (1 x phosphate buffered saline (PBS), 1% paraformaldehyde, ddH_2_O). Crosslinking took place in a vacuum desiccator (~ - 950 Millibars) for 15 minutes. 2,5 ml of the solution was replaced by 2,5ml of 1.25 M glycine in ddH_2_O, and placed in the vacuum for additional five minutes. Crosslinking solution was removed, and samples were washed three times in ddH_2_O. Tissues were removed from the bags, dried with paper towels and placed into new 50 ml falcon tubes. Tubes were snap-frozen and stored in -80°C till further processing. Chromatin immunoprecipitation was conducted by Diagenode ChIP-seq Profiling service (Diagenode Cat# G02010000) according to the company’s guidelines using the antibody directed against H3K4me3 (Diagenode, Belgium, cat. No. C15410003).

#### 2.2.2 Alignment and peak calling

**Figure 1:**
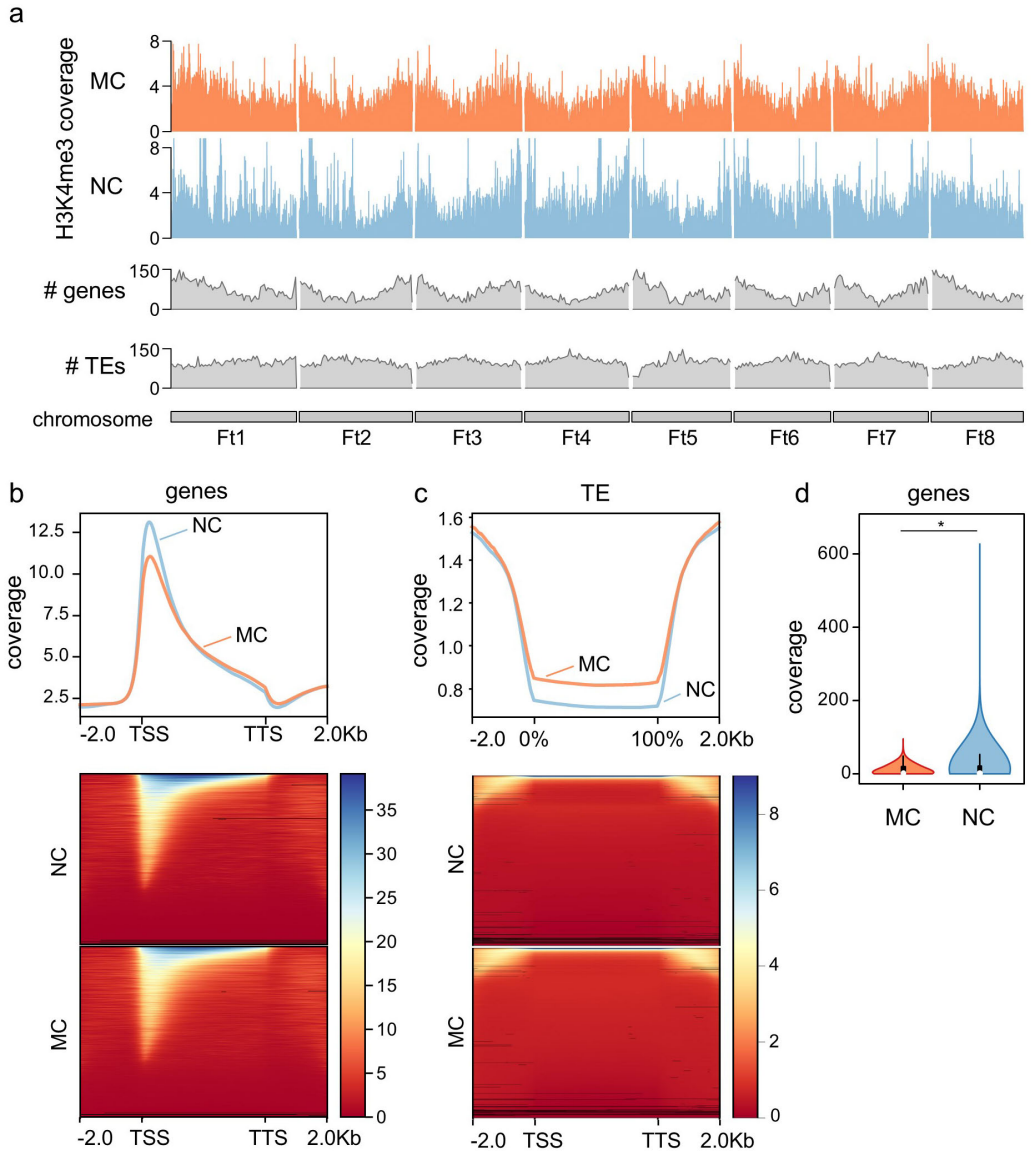
Genome-wide H3K4me3 distribution. **(A)** Chromosomal distribution of H3K4me3 in MC and NC, including genes and transposons (TEs) number along the chromosomes. **(B)** Metagene plot and heatmap of the distribution of H3K4me3 along genes, including -2.0 kb upstream the transcription start site (TSS) and +2.0 kb from the transcription termination site (TTS). **(C)** Metagene plot and heatmap of the distribution of H3K4me3 along TEs, including -2.0 kb upstream the TE body and +2.0 kb from the end of the TE. **(D)** Levels of H3K4me3 at the first 10% of the gene in MC and NC. Asterisk denote Mann–Whitney U test significant difference.

Data Analysis was performed by Diagenode Bioinformatics service (Diagenode Cat# G02010107). Sequencing was performed in paired-end 50 bp on an Illumina Novaseq, running Novaseq Control Software 1.7.0. Quality control of sequencing reads was assessed using FastQC v0.11.9 (Andrews, 2010). Trimming of the adaptors was done with cutadapt v3.5 (Krueger, 2024). Trimmed reads were aligned to the reference genome (*Fagopyrum_tataricum*) obtained from https://www.mbkbase.org/Pinku1/ (Zhang et al., 2017) using BWA software v.0.7.5a (Li and Durbin, 2009). PCR duplicates and multimapping reads were removed using samtools v1.15 (Li et al., 2009). Alignment statistics are collected in the **Supplementary Table S1**. Alignment coordinates were converted to BED format using BEDTools v.2.17 (Quinlan and Hall, 2010) and BED files were visualized by using the Integrated Genomic Viewer v2.17.4 (Robinson et al., 2011). Peak calling that corresponds to H3K4me3 enriched regions was performed using epic2 v0.0.52 (Stovner and Saetrom, 2019) with optimized peak calling parameters for histone modifications (undisclosed). General peak calling statistics are compiled in the **Supplementary Table S2**. The BED files were processed with deeptools2 v3.5.4 (Ramirez et al., 2016) to generate BigWig files of several bin size (10bp for **Figures 1A, B**, 50kb for **Figure 1C**). ThenkaryoploteR v.1.30.0 (Gel and Serra, 2017) was used to plot the density of H3K4me3 along genes and TE and across the chromosomes.

#### 2.2.3 Differential enrichment analysis

**Figure 2:**
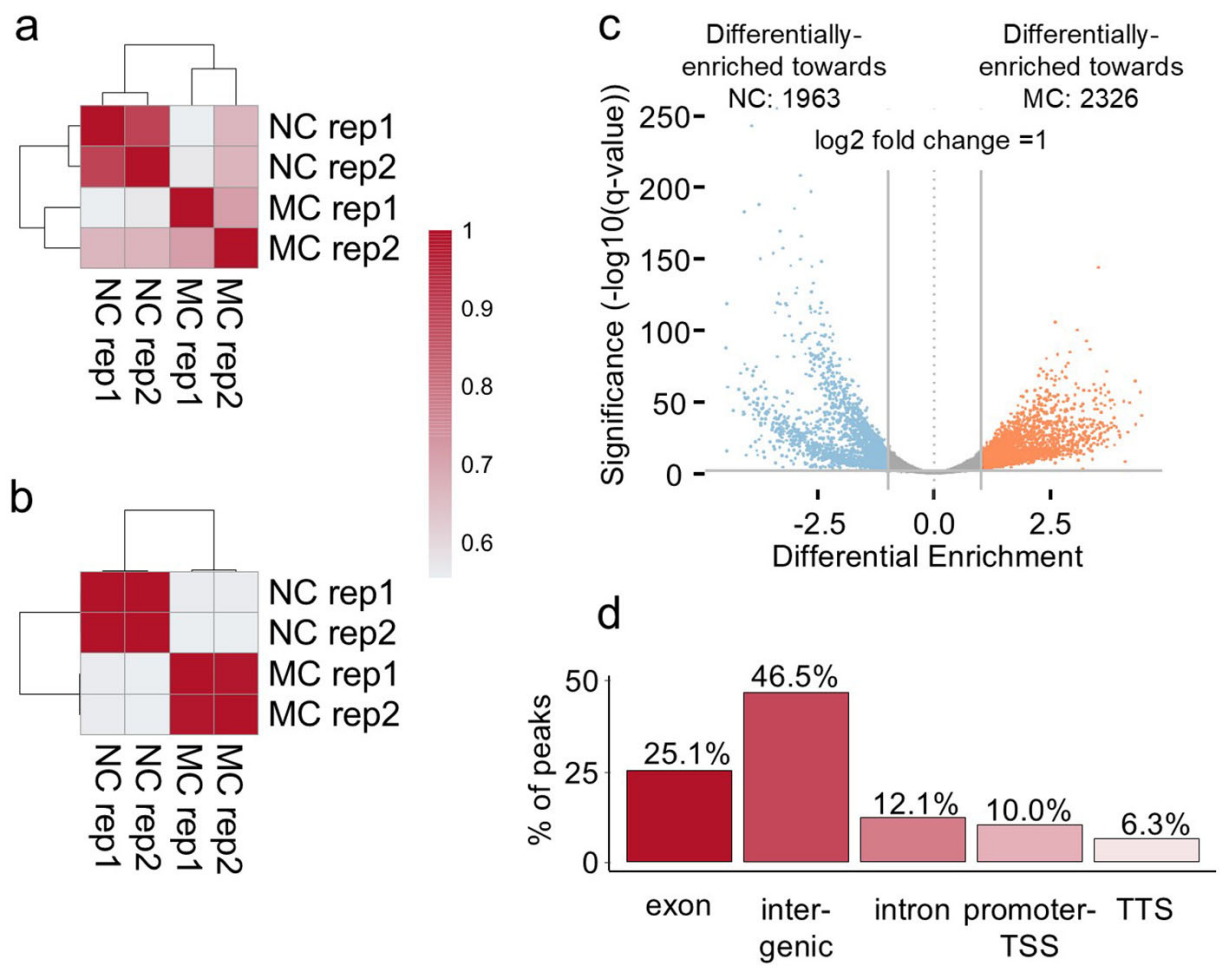
H3K4me3 differential enrichment of MC versus NC on day eleven of cultivation. **(A)** Pearson correlation on peaks detection of the MC and NC replicates. **(B)** Pearson correlation on peak enrichment of the MC and NC replicates. **(C)** Volcano plot of the differentially-enriched regions between MC and NC. The plot represents those 7372 differentially methylated regions, 4289 statistically significant regions with a two-fold change higher than one. 1963 regions displayed higher intensity in the NC (blue), whereas 2326 regions in the MC (orange). Regions in grey represent the peaks that are not passing the thresholds. The X-axis represents the log-fold differential enrichment. Vertical lines represent the threshold of the log2 change. The Y-axis represents the significance at 0,01. In this plot, each point represents a genomic region identified as a binding site (peak). The plot also gives information on whether there is a global increase or reduction of binding affinity in the first or second group of the comparison by evaluating the significant data points in the positive or negative Log Fold Change. **(D)** genomic distribution of the differentially enriched 4289 regions (considering a log2 fold change 1 and an adjusted p-value < 0.01 per annotation genomic features).

**Figure 3:**
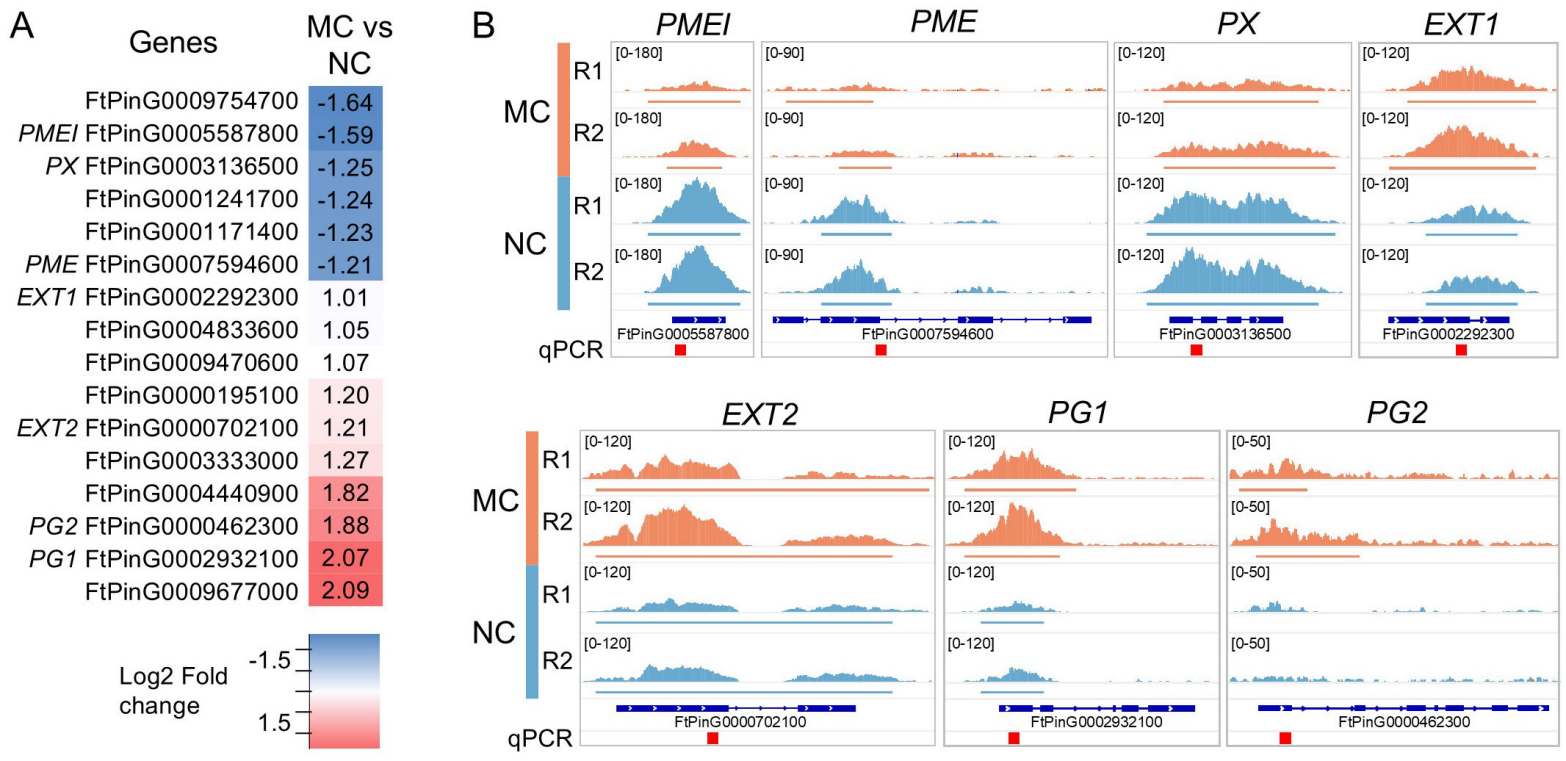
H3K4me3 in cell-wall related genes. **(A)** List of genes that presents a significantly enriched region (defined in **Figure 1**) in proximity of their TSS. Fold change (log2) is represented and names of the genes showed in **(B)** are indicated. The details of these genes can be found in the **Supplementary Table S9**. **(B)** Visual representation of the H3K4me3 enrichment on selected genes. Coverage of the replicas are represented in the same scale for each gene. Defined peaks are indicated below the coverage. Regions amplified by qPCR (**Figure 2**) are indicated as red square on the bottom. The Integrative Genomics Viewer was used for representation.

The differential enrichment quantitative analysis was performed with the R/Bioconductor package DiffBind (Stark and Brown, 2011; Ross-Innes et al., 2012). Difference in read concentration between MC and NC H3K4me3 peaks was estimated by comparing read densities computed over consensus peaks (peaks present in both replicates) (**Figures 2A, B**). The list of differentially enriched peaks between the two comparative groups is included in the **Supplementary Table S3**. Identified peaks were represented by Volcano plots in order to visualise which data points are differentially enriched (**Figure 2C**). Gene ontology analysis was performed by annotation of the genes with InterProScan 5.69-101.0 (Jones et al., 2014) and GO Enrichment implemented in Galaxy Australia version 21.09 (**Figures 3A, B**; Community, 2024).

### 2.3 Native chromatin immunoprecipitation

Chromatin immunoprecipitation experiments were carried out in four biological replicates, MC and NC samples were independently harvested on days two and six. A graphical depiction of the experiment was created with BioRender.com and is included in **Supplementary Figure S2** (the confirmation of publication and licensing rights is included as the **Supplementary Figure S9**). The protocol employed in our experiment was according to Trejo-Arellano et al. (2017) with minor modifications. For this procedure one gram of callus tissue was ground with liquid nitrogen using a pestle and mortar. Trejo-Arellano et al. (2017) used 400 mg of the tissue; however, in our case, using over two times more of the starting material was necessary due to the high vacuolisation of callus cells. The ground powder was suspended in 10 ml Honda buffer (0.5% Triton X-100, 0,4 M Sucrose, 25mM 2-amino-2-(hydroxymethyl)-1,3-propanediol (Tris) pH 7.4, 10 mM MgCl_2_, 2,5% Ficoll 400, 5% Dextran T40, 10 Mm β-mercaptoethanol, ddH_2_O) and placed on a rotator for 15-minute incubation in 4°C. Subsequently, the mixture was filtered twice through the miracloth, the first time with one layer and the second with two layers. Next, the tubes were centrifuged at 1500 g, 4°C for 5 minutes and the supernatant was discarded. The pellet was resuspended in 2 ml MNase buffer [50 mM Tris pH 8, 10 M NaCl, 5 mM CaCl_2_ and protease inhibitors (Aprotinin, Antipain Pepstatin A, Leupeptin, Sigma-Aldrich, USA)] and centrifuged again at 1500 g, 4°C for 5 minutes. Trejo-Arellano et al. (2017) protocol used the protease inhibitor cocktail (Roche - cOmplete™ Protease Inhibitor Tablets, Sigma-Aldrich, USA), however in our experiment employment of protease inhibitors prepared freshly before each experiment ensured their efficacy. The supernatant was discarded, and pellet was resuspended in 100 µl MNase buffer. Samples were placed on the thermoblock set at 14000 rpm, 25°C for 5 minutes, and MNase (1 µl/50 µl) was added. Trejo-Arellano et al. (2017) protocol employs digestion at 37°C for 7 minutes, however for *F. tataricum* calli the best digestion effects were observed at lower temperature and shorter time. Digestion was quenched by adding 10 mM EDTA; samples were incubated on ice for 10 minutes, then centrifuged for 5 minutes at 2000g at 4°C. The supernatant was transferred to a fresh tube, and the pellet was resuspended in 100 µl S2 buffer (50 mM Tris pH 8, 100 mM EDTA and protease inhibitors), incubated for 30 minutes on ice and centrifuged 5 minutes at 2000 g at 4°C. Supernatants were combined, and the pellet discarded. To check the digestion efficiency, 40 µl of the aliquot was purified with a column DNA extraction kit and run on 1% agarose gel. DNA concentration of each sample was checked in the BioSpectrometer fluorescence (Eppendorf, Germany). The DNA concentration was adjusted to 15 µg/per sample, diluted in ChIP incubation buffer (1 M Tris pH 8, 0,5 M EDTA pH 8, 0,1% Triton X-100, 1 M NaCl and protease inhibitors) to obtain 350 µl of the solution sufficient for the immunoprecipitation (IP) by IgG and anti-H3K4me3 (150 µl per IP sample and 50 µl for the input sample). Input was removed and stored at 4°C till further processing. Samples were divided into separate Eppendorf tubes 150 µl each, and antibodies were added: 2 µl of rabbit IgG (Sigma-Aldrich, USA) and 1 µl of anti-trimethylation of histone H3 at lysine 4 (anti-H3K4me3, Sigma-Aldrich, USA). Tubes were incubated with gentle rotation overnight at 4°C. The next day, 8 μl/100 μl Protein-A Dynabeads (InvitroGen, USA) were washed with ChIP incubation buffer (with fresh proteinase inhibitors) on the Magnarack (InvitroGen, USA) five times. Subsequently, 12 μl of Dynabeads per sample were added, and the incubation continued for 90 minutes at 4°C. During incubation wash buffers were prepared: wash buffer 1 (25 mM Tris pH 8, 10 mM EDTA pH 8, 0,1% Triton X-100, 50 mM NaCl), wash buffer 2 (25 mM Tris pH 8, 10 mM EDTA pH 8, 0,1% Triton X-100, 100 mM NaCl) and wash buffer 3 (25 mM Tris pH 8, 10 mM EDTA pH 8, 0,1% Triton X-100, 150 mM NaCl). In the Trejo-Arellano et al. (2017) protocol protease inhibitor cocktail was used also in the above buffers, however we found adding protease inhibitors at this step was unnecessary. After the incubation, the beads were washed twice in each wash buffer. Finally, one wash was performed with TE buffer (10 mM Tris pH 8, 1 mM EDTA pH 8). The beads were resuspended in 200 µl of freshly prepared ChIP elution buffer (1% SDS, 0.1M Na-HCO_3_) and incubated on a thermoblock at 65°C for 15 minutes with agitation. The supernatant was transferred to a fresh tube, and the beads were resuspended in 200 µl of ChIP elution buffer, and the incubation step was repeated. Supernatants were combined, beads were discarded. DNA purification of the input and ChIPed samples was performed by using phenol: chloroform. In the Trejo-Arellano et al. (2017) protocol, DNA elution and purification were done with IPure kit (Diagenode, Belgium), however for *F. tataricum* calli phenol:chloroform proved to be a more efficient method in terms of final quantity of recovered DNA.

### 2.4 Primer design, ChIP-quantitative PCR

Primers for ChIP were designed for selected regions on *F. tataricum* genome. The genomic sequence of *F. tataricum* cv. Pinku1 was downloaded from http://www.mbkbase.org/Pinku1/ (online access 4 February 2024) and loaded into Geneious 11.1.2 software (http://www.geneious.com (Kearse et al., 2012). Gene-specific primers for positive and negative H3K4me3 regions were designed using the program’s ‘design primers’ function. Primers used are listed in the **Supplementary Table S4**.

**Figure 4:**
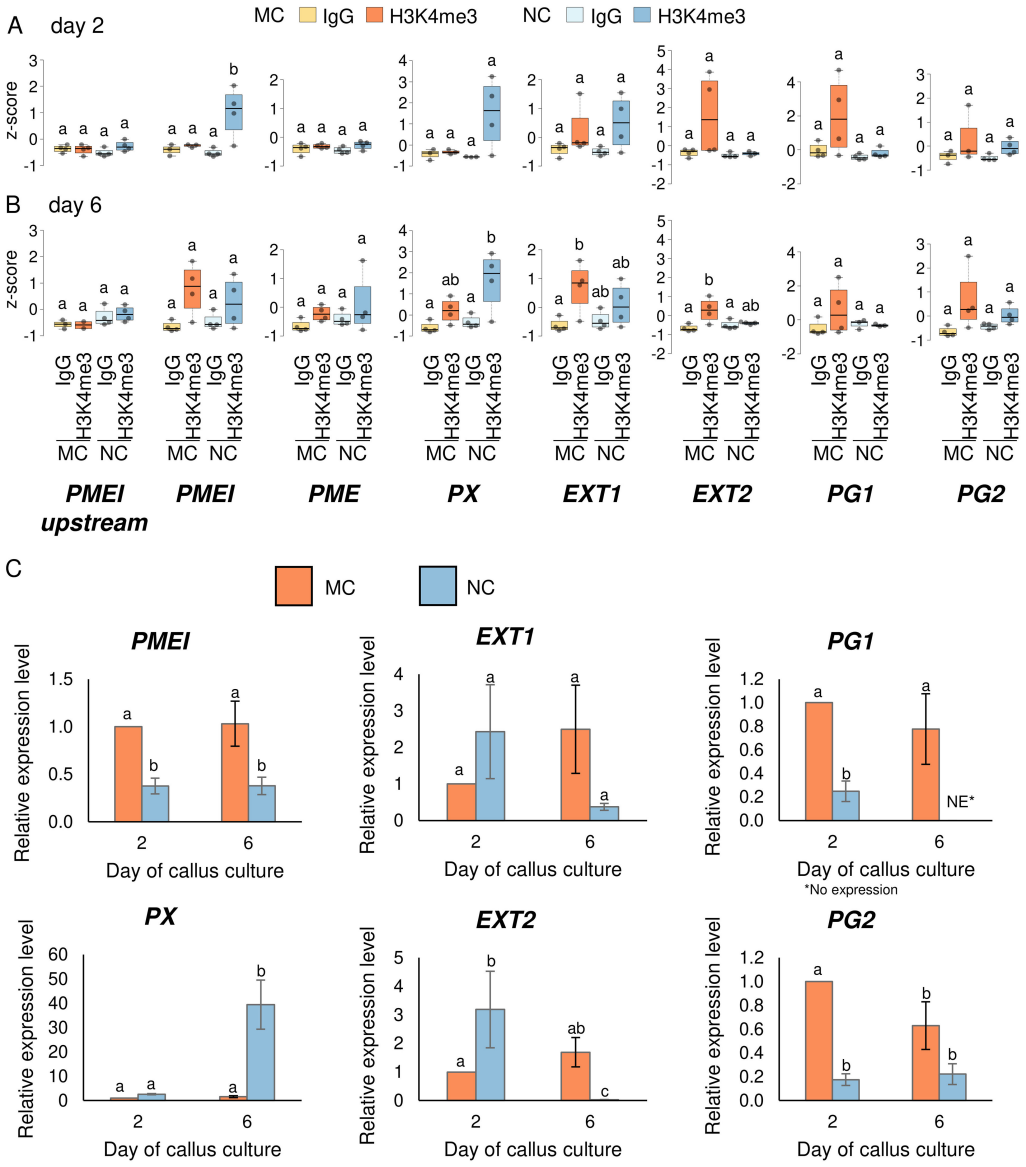
H3K4me3 enrichment in cell wall related genes at day two and six of the passage. ChIP-qPCR of H3K4me3 in MC and NC at day two **(A)** and day six **(B)** on selected genes. Box plots show the distribution of normalised z-scores of H3K4me3%Input of experiments performed simultaneously. One-way ANOVA followed by Tukey test was applied, and significance are marked by letters. **(C)** Relative expression level of *PMEI, PX, EXT1, EXT2, PG1, PG2.* The expression level of genes in MC and NC was normalized with the constitutive genes *ACTIN* and *SAND* and calibrated to expression on day two in MC. Different letters indicate a significant difference between the tissues and days according to Tukey’s HSD test (p < 0.05; n = 3; means ± SE are given).

Input and ChIPed DNA samples quantification was performed by quantitative PCR (qPCR) using the 480 LightCycler^®^ 480 SYBR Green I Master in a LightCycler^®^ 480 Real-Time PCR System (Roche, Germany) in two technical replicates. Quantitative cycles were obtained and % input was calculated. To represent the ChIP data, results obtained from four independent biological replicates were calculated as z-scores of the % input (**Figures 4A, B**) z-scores were used to minimize intraexperimental technical variability that was not possible to be avoided because the ChIP workload did not allow the simultaneous execution of all the immunoprecipitation reactions. Ct values are available in **Supplementary Table S5**.

### 2.5 Reverse transcription quantitative PCR

Total RNA was isolated from MC and NC on days two and six of callus culture. Total RNA was extracted using the FastPure Plant Total RNA Isolation Kit (Polysaccharides and Polyphenolics-rich) from Vazyme Biotech (Red Maple Hi-tech Industry Park, PRC). The RNA concentrations were quantified with a Nano-Drop ND-1000 spectrophotometer (NanoDrop Technologies, USA). To remove any residual DNA, the RNA samples were treated with an RNase-free DNase Set (Qiagen, Germany). Reverse transcription (RT) was carried out using oligo-dT primers and the Maxima H Minus First Strand cDNA Synthesis Kit (Thermo Fisher Scientific, USA). The resulting cDNA was diluted four-fold with water, and 2 µl of this diluted cDNA was used in the qPCR reactions. The qPCR was performed in a 10 µl reaction volume using the LightCycler^®^ 480 SYBR Green I Master mix (Roche, Switzerland). The same primers were used for ChIP-qPCR and RT-qPCR with exception of *PX*, which is annotated in the **Supplementary Table S4**. Constitutive genes *SAND* and *ACTIN* were used as previously (Sala-Cholewa et al., 2024). qPCR was conducted on a LightCycler 480 system (Roche, Switzerland) under the following cycling conditions: an initial denaturation at 95°C for 5 minutes, followed by 40 cycles of 95°C for 10 seconds, a primer-specific annealing temperature for 20 seconds, and 72°C for 10 seconds. For melt curve analysis, denaturation was performed at 95°C for 5 seconds, followed by 65°C for 1 minute, and a gradual increase to 98°C (0.1°C/s) for fluorescence measurement. Ct values were determined using LinRegPCR software (version 11, Academic Medical Centre, The Netherlands). The plant tissues for the RT-qPCR analysis were prepared in three biological replicates, with each biological replicate having two technical replicates. Relative expression levels were calculated using the 2^–ΔΔCT^ method, where ΔΔC_T_ is defined as ΔC_T_^reference condition^ − ΔC_T_^compared condition^. The one-way ANOVA (p < 0.05) followed by Tukey’s honestly-significant-difference test (Tukey HSD-test) (p < 0.05) were used to calculate any significant differences between the experimental combinations (**Figure 4C**). Ct values are included in the **Supplementary Table S6**.

### 2.6 Immunohistochemical analysis

The MC and NC were fixed overnight at 4°C in a solution containing 4% paraformaldehyde and 1% glutaraldehyde in PBS (pH 7.3). Following fixation, the samples underwent three 15-minute washes in PBS and were then subjected to a dehydration process using increasing concentrations of ethanol (10%, 30%, 50%, 70%, 90%, 100%, each twice) before gradual embedding in London Resin (LR White resin, USA). Subsequently, the embedded samples were sliced into 1.5 µm thick cross-sections using an EM UC6 ultramicrotome (Leica Biosystems, Germany), and placed on glass slides coated with poly-L-lysine. For immunocytochemical analyses, the sections were treated with a blocking buffer (2% foetal calf serum and 2% bovine serum albumin in PBS) for 30 minutes at room temperature (RT). Next, the sections were incubated overnight at 4°C with primary monoclonal antibodies (Plant Probes, UK) diluted 1:20 in a blocking buffer. The antibodies used are listed in **Supplementary Table S7**. After three washes in the blocking buffer, the sections were incubated with a secondary antibody, AlexaFluor 488 goat anti-rat IgG (Jackson ImmunoResearch Laboratories, UK), diluted 1:100 in the blocking buffer for two hours at RT. Following another wash in the blocking buffer and rinsing three times in PBS, the sections were stained with calcofluor white (Sigma-Aldrich, USA) in PBS to visualise cell walls. After rinsing in PBS and distilled water, the dried sections were mounted on slides using Fluoromount (Sigma-Aldrich, USA).

As a negative control, the primary antibody was omitted, and the blocking buffer was applied along with all other procedure steps. Observations were performed, and the images were taken with an AxioImager Z2 epifluorescence microscope equipped with an AxioCam Mrm monochromatic camera (Zeiss, Germany) equipped with narrow-band filters for AlexaFluor 488 and DAPI.

## 3 Results

### 3.1 Morphology of *F. tataricum* morphogenic and non-morphogenic calli

A detailed description of the callus tissue morphology used in this research is available in our previous publications (Betekhtin et al., 2017, 2019; Tomasiak et al., 2023). The morphology of MC and NC is included in **Supplementary Figure S1**. Morphogenic callus (MC) comprises of proembryogenic cell complexes (PECCs) and soft callus cells (SCCs) (**Supplementary Figure S1A**, white and black asterisks, respectively). NC comprises solely of highly vacuolated, parenchymatous cells (**Supplementary Figure S1B**). Transfer to a fresh medium, which contains high auxin concentration, triggers PECCs disintegration, accompanied by the release of secretion, leading to the formation of new PECCs in the SCCs (between the 6^th^ and 11^th^ days of cultivation) (Rumyantseva et al., 2003; Betekhtin et al., 2017). Because the changes present during MC’s development are major, day two was selected as a point where PECCs disintegrate, whereas day six was a point where subsequent new PECCs reinitiation processes begin. On day eleven of the culture, MC exhibits high content of embryogenic cells, whereas NC’s parenchymatous cells characterise with high accumulation of the oxidative stress. Moreover, as demonstrated by our previous research, day two and day six exhibited the biggest changes in the global levels of H3K4me3 in MC and NC during the passage (Tomasiak et al., 2023).

### 3.2 H3K4me3 levels in MC and NC calli

Calli of *F. tataricum* with varying morphogenic capacities exhibited global epigenetic changes as demonstrated by fluctuations in DNA methylation and histone modifications (Tomasiak et al., 2023). To have a more precise picture of these changes, this work focused on the analysis of the genomic H3K4me3 mark distribution in MC and NC by performing a ChIP-seq in both callus types on day eleven of cultivation. The distribution of H3K4me3 along the *F. tataricum* chromosomes (Ft1-Ft8) (**Figure 1**) shows that the mark in both MC and NC is distributed along the chromosomes, with a pattern that follows gene distribution, and contrary to the transposable elements (TEs), indicating that H3K4me3 is distributed in the transcriptional active chromatin (where genes are located) and reduced in the silent chromatin (where enrichment of TEs occurs). In fact, when the H3K4me3 levels in both NC and MC are plotted on genes, the mark appears to be highly enriched, with clear higher levels of the mark at the transcriptional start site (TSS) (**Figure 1B**). On the contrary, H3K4me3 is absent in repetitive elements such as TEs (**Figure 1C**), as expected with a mark related to genes. As can be noted from the metagene plot (**Figure 1B**), the levels of H3K4me3 in the TSS are higher in NC than in MC. Accordingly, when we compared the coverage of H3K4me3 in the first 10% of the gene body, a significant increase of the mark was found in the NC, likewise the global content of the mark which is higher in NC than MC (**Figure 1D**) as previously reported by immunocytochemical analysis (Tomasiak et al., 2023).

Further analysis was conducted and H3K4me3 peaks were defined. Based on the detected peaks, the Pearson correlation between the samples analysed indicates that MC replicates and NC replicates form clusters together, both when peak location (**Figure 2A**) or the intensity of the peaks located at the same place (**Figure 2B**) were compared. The proper separation of the clustering demonstrates that MC and NC comparison presents a significant statistical difference in the peak intensity. By a differential analysis, 7372 regions were detected to be statistically different (adjusted p-value (q-value or FDR) < 0.01) (**Figure 2C**; **Supplementary Table S3**). Of those regions, 4289 not only statistically significant but also presented a log2-fold change higher than one. This cutoff signifies that the difference between the peak intensities is higher than the fold change of two. 1963 regions displayed higher intensity in the NC (blue), whereas 2326 regions in the MC (orange) (**Figure 2C**). The genomic distribution of these 4289 differential enriched regions (**Figure 2D**) shows that the majority of the regions were associated with exons and intergenic regions, with a lower amount of regions associated with introns and transcriptional termination site (TTS). Surprisingly, the promoter and TSS represent only 10.03% of those regions. However, from the regions classified as exon, 61% of them located in the first exon. Based on the distribution of the mark peaking at the TSS, we expect that the differentially enriched regions classified as promoter-TSS and first exon have the most important role in regulation of the gene expression. For that reason, we analysed the genes whose TSS was in close proximity (less than 1kb) to the differentially enriched regions. We obtained 1709 genes; in 1057 genes H3K4me3 was higher in MC and in 652 genes higher methylation prevailed in NC. A gene ontology analysis of these genes gave only significant enrichment on the list of genes with higher levels of the mark in MC (**Supplementary Table S8**). Many categories were photosynthesis-related activities (such as the photosynthesis itself, heme binding, oxidoreductase activity, iron ion binding), and some to metabolic pathways (glycogen (starch) synthase activity, UDP-glycosyltransferase activity) and the membrane (as cellular component category).

### 3.3 Comparison of H3K4me3 levels on cell wall related genes during the MC and NC growth

As cell wall phenolic composition in NC has been reported to be lower than in MC (Akulov et al., 2018), we explored among the genes that had in proximity of the TSS a differentially enriched region (log2 FC < 1 and an adjusted p-value < 0.01), those related to cell wall (**Figure 3A**). We found that 16 genes presented differential methylation, 6 of them with enrichment towards NC and 10 towards MC, (**Supplementary Table S9**). Among the cell wall related genes enriched in NC, we found two genes related to xyloglucan catabolism, two related to pectin (*PECTIN METHYLESTERASE INHIBITOR*, *PMEI* and *PECTIN METHYLESTERASE*, *PME*), and a peroxidase (*PEROXIDASE*, *PX*). Polygalacturonase was also found, and two other *POLYGALACTURONASES* (named as *PG1* and *PG2*) were found as enriched in MC. Pectin-related genes were also found among the H3K4me3-enriched in MC, but some other gene categories were specific, such the wall-associated receptor kinases and *EXTENSINS* (named as *EXT1* and *EXT2*).

Seven genes of these cell wall proteins were selected to explore their H3K4me3 levels by N-ChIP-qPCR (**Figures 4A, B**) and relative expression by RT-qPCR (**Figure 4C**) at two earlier time points of the culture: day two, when PECCs disintegrate, and day six, when new PECCs are formed. From the genes that were enriched in H3K4me3 in NC at day eleven (**Figure 3A**), *PMEI*, *PME* and *PX* were studied. *PMEI* showed significant enrichment of this mark in NC as early as day two. Despite the H3K4me3 levels on day six showed a certain enrichment compared to an H3K4me3-negative region upstream of the gene (**Figures 4A, B**; **Supplementary Figure S3** for primer location and two other negative regions tested), this difference was not statistically significant compared to the basal levels (IgG signal). Although the H3K4me3 mark has been associated with higher transcriptional activity, expression results showed statistically lower levels of expression in NC on both, day two and day six, despite higher methylation. The *PME* gene was not enriched in H3K4me3 in either NC or MC on day two or day six, suggesting that NC methylation may occur after these time points, as this mark was present on day eleven. According to this low methylation, expression levels of *PME* were below the limits of detection by RT-qPCR (**Supplementary Table S6**). For *PX*, H3K4me3 were significantly higher only in NC on day six. In this case, the relative expression of *PX* positively correlated with methylation, as expression in NC on day six increased significantly, reaching a level 39.4 times higher than in MC on day two. From the genes that showed higher levels of H3K4me3 in MC than in NC on day eleven (**Figure 3A**), *EXT1*, *EXT2*, *PG1* and *PG2* were analysed. For *EXT1* and *EXT2*, no significant differences in H3K4me3 were observed on day two. A significant increase was only observed in these genes in MC on day six when compared to basal levels (IgG). Therefore, as with *PME*, levels of methylation on day eleven may have been stablished by day six. For *EXT1*, relative gene expression remained stable across the two time points and in the different callus type, while *EXT2* showed higher expression in NC compared to MC on day two, but lower expression in NC on day six (with a decrease being 41.7 times lower than in MC at day two). As with *PMEI*, expression on *EXT2* did not correlate with H3K4me3 levels. The enrichment of H3K4me3 in *PG1* and *PG2* across the examined days did not show statistically significant differences. The expression of *PG1* was significantly higher in MC than in NC on both culture time points (with expression levels below the technical limits on day six in NC). Similarly to *PG1*, in *PG2*, relative expression in MC was higher than in NC, but this occurred only on day two. The accumulation of H3K4me3 observed in *PG1* and *PG2* within MC, even though it was not statistically significant, points to a potential role for this modification in sustaining higher expression.

Altogether, the dynamic regulation of H3K4me3 methylation across different time points and callus types indicates a complex relationship between epigenetic modifications and gene expression in cell wall-related genes. While some genes, such as *PMEI* and *PX*, show a certain correlation between increased H3K4me3 levels and higher expression, others, like *PME* and *EXT2*, do not follow this pattern, suggesting that additional regulatory mechanisms may be involved.

### 3.4 Immunodetection of pectic and extensin epitopes

The specific cell wall epitope distribution was analysed on day two and six of the passage, corresponding to the days when H3K4me3 ChIP was conducted. Five different antibodies were applied using an immunocytochemical approach. Four were against different pectic epitopes (LM5, LM6, LM19 and LM20) and one against the extensin (JIM20). Results are summarised in **Table 1**, and the images from the immunolocalisation are included in **Supplementary Figures S4**–**S8**. Separating the fluorescence signals in the cell wall and the plasma membrane has proven difficult when using light microscopy. Therefore, those two compartments are described together.

**Table 1:** Summary of the immunocytochemical detection of selected epitopes in morphogenic and non-morphogenic callus (positive (+) or negative (-) reaction).

			Morphogenic callus (MC)			Non-morphogenic callus (NC)		
			Cell wall compartments	Internal cell compartments	Callus surface	Cell wall compartments	Internal cell compartments	Callus surface
**Pectins**	**LM5**	Day 2	**+**	**-**	**-**	**+**	**-**	**-**
		Day 6	**+**	**-**	**-**	**+**	**-**	**-**
	**LM6**	Day 2	**-**	**-**	**-**	**-**	**-**	**-**
		Day 6	**-**	**+**	**-**	**-**	**-**	**-**
	**LM19**	Day 2	**+**	**+**	**+**	**+**	**+**	**+**
		Day 6	**+**	**+**	**+**	**+**	**+**	**+**
	**LM20**	Day 2	**+**	**-**	**+**	**+**	**+**	**+**
		Day 6	**+**	**-**	**+**	**+**	**+**	**+**
**Extensin**	**JIM20**	Day 2	**-**	**-**	**-**	**-**	**-**	**-**
		Day 6	**-**	**-**	**-**	**-**	**-**	**-**

Four antibodies were employed to analyse the pectin epitopes: LM5, LM6, LM19, and LM20. LM5 signal was present in MC and NC on each examined day in the cell wall but absent in the cell internal compartments and on the surface (**Supplementary Figure S4**). LM6, on the other hand, in MC was not present at the beginning of the passage, it appeared only on day six solely in a dotted manner in the inner cell compartments (**Supplementary Figures S5A, B**, b2 white arrows). In NC, this epitope was absent regardless of the day of the passage (**Supplementary Figures S5C, D**). LM19 was present both in MC and NC on all examined days, and all examined compartments and the callus surface (**Supplementary Figure S6A, B**, white arrows; d2 and d2’ inset; d3 and d3’ inset). LM20 was present in the MC across the passage in the cell wall and on the callus surface. In NC, LM20 was present not only in the cell wall and on the surface (**Supplementary Figure S7**; c2 and c2’ inset; c3 and c3’ inset) but also in the internal cell compartments (**Supplementary Figure S7** d2, d3). The immunocytochemical analysis included one antibody against the extensins (JIM20); however, neither in MC nor NC was the reaction positive (**Supplementary Figure S8**).

## 4 Discussion

This research aimed to analyse and compare the enrichment of histone H3 at lysine K4 trimethylation (H3K4me3) in the genes coding for the cell wall proteins in *F. tataricum* MC and NC. To achieve this objective, the ChIP protocol was optimised to be applicable for *F. tataricum* callus tissue. To this date, a verified ChIP method has not been reported for *Fagopyrum* species, possibly due to its high content of phenolic compounds, which may affect immunoprecipitation efficiency. In plants, X-ChIP has been used, in some cases to address the role of histone marks in regulating the expression of genes related to cell wall biosynthesis. Experiments conducted on *Zea mays* demonstrated increased H3K9ac of two cell 5wall genes under the salt stress, suggesting that histone modifications may be important mitigating the harmful effects of excessive salinity on plants (Li et al., 2024). Native ChIP has not been commonly used in plant research (Ricardi et al., 2010; Huang et al., 2020). However, a few papers describe this method’s use for studying plant pathogenic fungi (Cosseau et al., 2009; Soyer et al., 2014, 2015). The N-ChIP method beats traditional methods in profiling histones and histone modifications due to its superior antibody specificity, higher pull-down efficiency, reduced background noise, and minimised bias towards open chromatin (O'Neill and Turner, 2003; Kasinathan et al., 2014), and it has been necessary in this work to be able to analyse *F. tataricum* callus material at early time points of the passage (day two and day six).

In this research, *F. tataricum* MC and NC samples underwent ChIP-seq to assess the global enrichment of H3K4me3. As a result, we were able to notice H3K4me3 distribution clearly differs between MC and NC (**Figures 1**, **2D**), indicating that the enrichment of H3K4me3 could affect transcription potential of many different genes between those two tissue types. This link of H3K4me3 and transcriptionally active regions in *F. tataricum* is supported by the mark presence on genes and the strong enrichment around the TSS (**Figure 1A**). Available literature coincided with these results. A ChIP-seq analysis of rice callus demonstrated that H3K4me3 peaks are predominantly associated with gene regions, particularly near TSS, indicating its role in active transcription (Zhao et al., 2020). Research on *Eucalyptus grandis* showed that the vast majority of H3K4me3 peaks were gene-associated, particularly near exons and introns. It was also shown that H3K4me3 was present at several highly expressed genes involved in the secondary cell wall (Hussey et al., 2015).

Research demonstrated that the transcriptome analysis in the passage dynamics of soybean revealed active regulation of genes involved in cell wall modification. Those genes’ expression proved to be essential for callus induction and proliferation. Researchers concluded that cell wall related genes, such as those responsible for cell wall degradation and synthesis, are differentially expressed during various stages of callus formation, indicating an intricate regulatory mechanism that includes H3K4me3 (Park et al., 2023). H3K4me3 has an indispensable role in maintaining the plasticity and dynamic nature of callus tissues, enabling the reprogramming of somatic cells into a pluripotent state capable of regeneration​ (Foroozani et al., 2021).

In this work, ChIP-seq H3K4me3 analysis on day eleven of cultivation acted as a reference point for further analysis. This led to selecting the genes coding for *PECTIN METHYLESTERASE INHIBITOR (PMEI), PECTIN METHYLESTERASE (PME)*, *PEROXIDASE (PX)*, two different regions coding for *EXTENSINS (EXT1, EXT2)*, and two regions coding for *POLYGALACTURONASES (PG1, PG2)*. *PMEI* and *PME* presented lower levels of H3K4me3 in MC on day eleven. *PME* did not show changes of methylation before day eleven, and its expression was undetectable. For *PMEI*, levels of H3K4me3 on day two were already elevated in NC. Despite this methylation pattern, expression in NC was lower than in MC. This suggests an increase in the activity of this enzyme during the MC’s PECC disintegration. In *Arabidopsis*, the expression of *PMEI* genes has been linked to enhanced shoot regeneration from callus tissues. The maintenance of a higher degree of pectin methylestrification by PMEIs supports the structural changes required for the formation of new shoots from callus cells (Cao et al., 2021). Additionally, by regulating the activity of PMEs, PMEIs regulate the level of pectin methylesterification, which is crucial for maintaining cell wall plasticity. It is essential for the callus’ cells to undergo the modifications which are subsequently required for differentiation and development into specialised tissues with morphogenic potential (Zhang et al., 2024). Our immunohistochemical research revealed differences in the distribution pattern of low (LM19) and high methyl-esterified homogalacturonan (HG; LM20) that are strongly connected with PME activity. LM19 was present in all examined compartments and on the callus surface in MC and NC across the passage. LM20, on the other hand, was only absent in the internal cell compartments in MC. It has been shown that a reduction in wall stiffness was associated with increased pectin demethylesterification modulated by PME (Wormit and Usadel, 2018). The occurrence of both forms of HG in callus cells has been previously observed for other species in both monocots (Betekhtin et al., 2016, 2018) and dicots (Kuczak and Kurczynska, 2020; Popielarska-Konieczna et al., 2020). This may indicate that the presence of high and low methylesterified HG, and therefore the effects of PME and PMEI, play an important role in developmental processes in callus cells. Aside from differences in the occurrence of HG epitopes, our observations showed the presence of LM5 epitope in the cell wall compartments in MC and NC, whereas LM6 was present on days six of the passage in MC but completely absent in NC. Antibodies LM5 and LM6 recognise carbohydrate residues within the galactan and arabinan side chains of rhamnogalacturonan I (RG-I), respectively (McCartney et al., 2000). These two forms of RG-I side chains show variable occurrence in cell walls and may be modified depending on both biotic and abiotic factors (Jaskowiak et al., 2019). Moreover, walls rich in galactan are strong and stiff, while those with arabinan predominance are flexible (Schols and Voragen, 1996). Previous immunocytochemical studies of *in vitro* cultures showed the presence of the LM5 epitope, especially in non-embryogenic cells, as well as a poor amount of LM6 epitope (Xu et al., 2011; Pilarska et al., 2013; Potocka et al., 2018), which is in accordance with the presented results.

Peroxidases facilitate the cross-linking cell wall polysaccharides and glycoproteins, increasing the cell wall’s mechanical strength. This process is vital for stabilising callus cells and promoting their differentiation into specialised tissues. They are involved in the biosynthesis and polymerisation of lignin, especially during callus differentiation, which is crucial for the structural development of new tissues. *PX* enrichment in H3K4me3 is on an overall low level across the passage in MC with the course of the passage, in correlation with lower gene expression when the new PECCs arise. In peony callus, two peroxidase genes were found to be significantly down-regulated in callus with a high differentiation rate relative to callus with a low differentiation rate (Zhu et al., 2022). In NC, on the other hand, the high levels of H3K4me3 and increased relative expression of *PX* (~40 times) on the sixth day of the passagemay be involved in the rapid proliferation of this tissue.

Polygalacturonases (PGs), along with other cell wall-modifying enzymes, are part of a complex network that regulates the expression of genes involved in cell wall biosynthesis and modification. This network ensures that the cell wall remains dynamic and responsive to the needs of the differentiating cells. In *F. tataricum, PG1* and *PG2* are enriched in H3K4me3 in MC but not in NC on day eleven, suggesting active expression of those genes during cellular redifferentiation. Accordingly, gene expression of *PG1* and *PG2* on day two and six is higher in MC, also in agreement with a certain accumulation of H3K4me3 observed within MC, even though it is not statistically significant. Studies have shown that manipulating *PG* expression can influence the efficiency of callus formation and subsequent regeneration. For example, overexpression of *PG* genes can lead to enhanced callus proliferation and improved shoot regeneration from callus tissues​ (Zhang et al., 2024).

Obtained results suggest that the acquisition of embryogenic potential requires a complex and fine-tuned coordination between multiple processes and involves multiple components in the cell wall. Using two types of *F. tataricum* callus with different capacity for morphogenesis allowed us to conduct a comparative analysis of the H3K4me3 influence on gene expression coding for cell wall genes during the course of callus development.

## 5 Conclusions

The research aimed to analyse and compare the enrichment of H3K4me3 in genes coding for cell wall proteins in *F. tataricum* MC and NC. The study also optimised the N-ChIP protocol for *F. tataricum* callus tissue, a challenging task due to the species’ high phenolic content, which can hinder immunoprecipitation efficiency. ChIP-seq data revealed that H3K4me3 enrichment differs significantly between MC and NC. Further analysis revealed a higher enrichment of H3K4me3 near the TSS, indicating the importance of H3K4me3 on transcriptionally active regions. Additional ChIP-qPCR analysis showed that genes coding for *PME*, *PMEI*, *PX, EXTs* and *PGs* settled their enrichment to some extent in MC or NC already at day six or even day two of the passage, suggesting these genes became actively transcribed during callus differentiation and morphogenesis, and that these processes are highly dynamic. The increase in the relative expression of *PX* on day six in NC suggests that this gene is involved in the rapid proliferation of cells in NC.

In summary, this study provides novel insights into the epigenetic regulation of callus development in *F. tataricum*, highlighting the crucial role of H3K4me3 in activating genes involved in cell wall biosynthesis and modification, which are essential for the morphogenesis and regeneration of plant tissues. Our study offers new insights into the epigenetic mechanisms driving the cell wall composition during callus development in *F. tataricum*, offering valuable information in acquiring embryogenic potential.

## Data Availability

The data presented in the study are deposited in the NCBI GenBank BioProject, accession number: PRJNA1142539.

## Supplementary material

Supplementary Figure 1 Morphology of the *F. tataricum* calli. **(A)** morphogenic callus (MC); soft callus (black asterisks) and newly formed proembryogenic cell complexes (white asterisks); **(B)** non-morphogenic callus. Scale bar: 0.5 cm.

Supplementary Figure 2 Graphical depiction of N-ChIP protocol.

Supplementary Figure 3 H3K4me3 enrichment in negative control regions. **(A)** Visual representation of the H3K4me3 enrichment on negative control selected regions: *PME* upstream (**Figure 4**) and *S-ELF3*. Coverage of the replicas are represented in the same scale for each gene. Regions amplified by qPCR are indicated as red square on the bottom. **(B)** ChIP-qPCR of H3K4me3 levels of the negative regions around the *S-ELF3* gene. Box plots show the distribution of normalised z-scores of H3K4me3%Input for MC and NC across the time points (day two and day six).

Supplementary Figure 4 Immunocalisation of LM5 in *F. tataricum* MC and NC in passage dynamics, i.e., day two and day six. *FB* fluorescent brightener. Scale bar: 10 μm.

Supplementary Figure 5 Immunocalisation of LM6 in *F. tataricum* MC and NC in passage dynamics, i.e., day two and day six; white arrows- signal in the inner cell compartments. *FB* fluorescent brightener. Scale bar: 10 μm.

Supplementary Figure 6 Immunocalisation of LM19 in *F. tataricum* MC and NC in passage dynamics, i.e., day two and day six; signal on the callus surface:d2 and d2’ inset; d3 and d3’ inset. *FB* fluorescent brightener. Scale bar: 10 μm.

Supplementary Figure 7 Immunocalisation of LM20 in *F. tataricum* MC and NC in passage dynamics, i.e., day two and day six; signal on the callus surface: d2 and d2’ inset; d3 and d3’ inset. *FB* fluorescent brightener. Scale bar: 10 μm.

Supplementary Figure 8 Immunocalisation of JIM20 in *F. tataricum* MC and NC in passage dynamics, i.e., day two and day six. *FB* fluorescent brightener. Scale bar: 10 μm.

Supplementary Figure 9 BioRender.com publication licence for **Supplementary Figure 1**; graphical depiction of N-ChIP protocol.
