# Supplementary figures and images for: H3K4me3 changes occur in cell wall genes during the development of Fagopyrum tataricum morphogenic and non-morphogenic calli
Source: Front Plant Sci. 2024 Sep 25;15:1465514. doi: 10.3389/fpls.2024.1465514 (PMC11461221; doi:10.3389/fpls.2024.1465514)

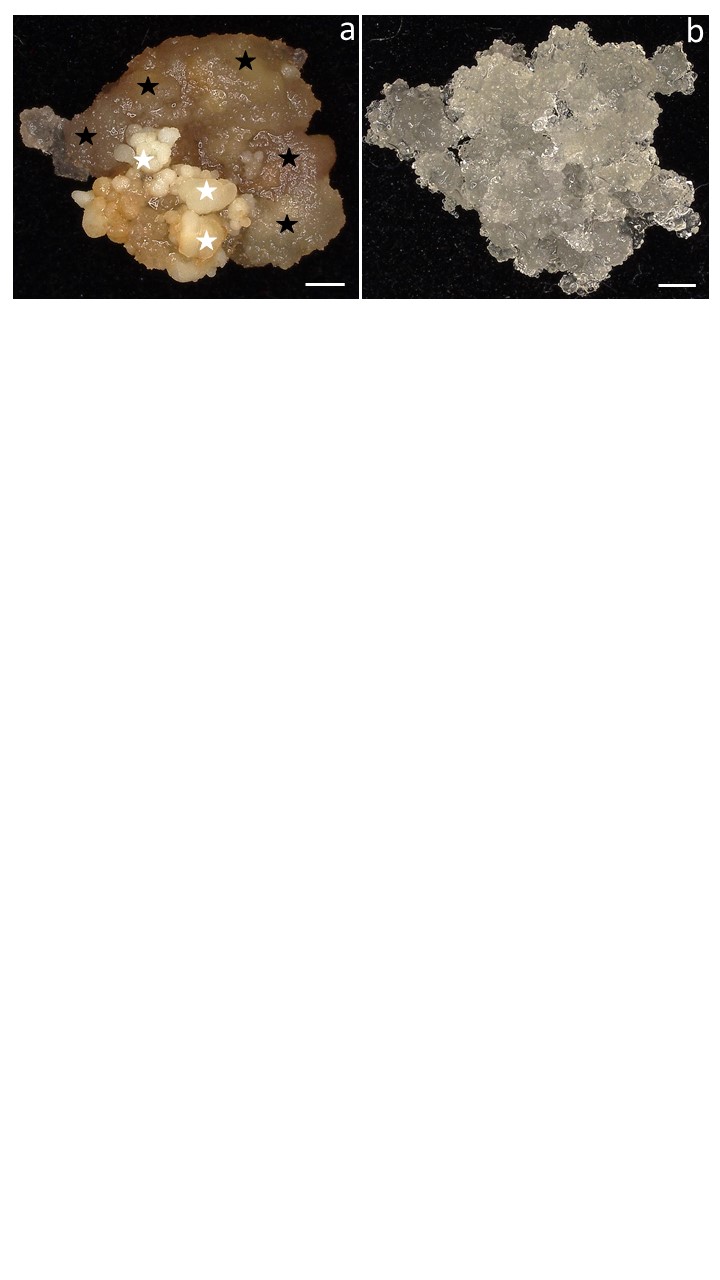

Supplement: Supplementary Figure 1 — Morphology of the F. tataricum calli. (A) morphogenic callus (MC); soft callus (black asterisks) and newly formed proembryogenic cell complexes (white asterisks); (B) non-morphogenic callus. Scale bar: 0.5 cm. [file Image1.jpeg]

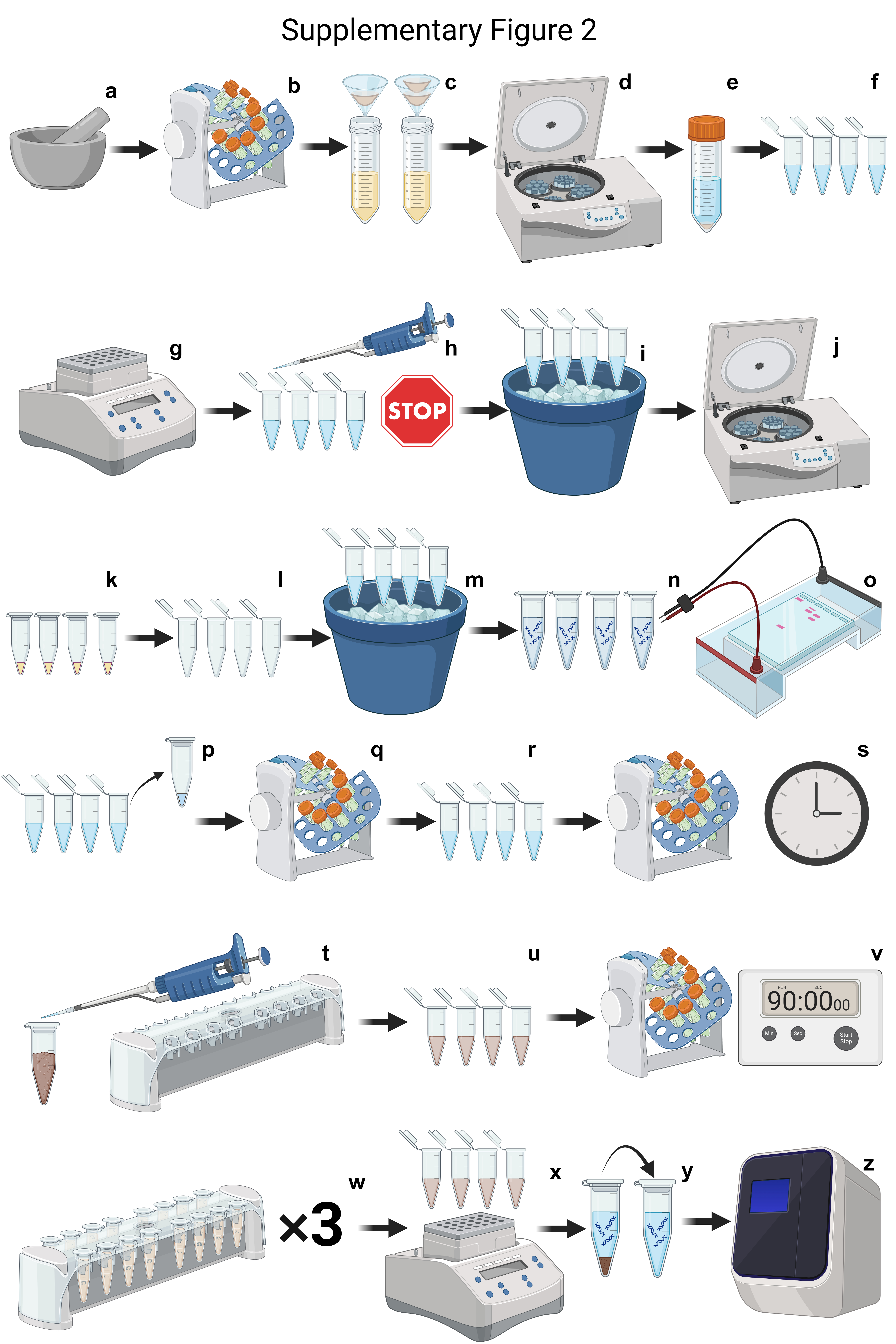

Supplement: Supplementary Figure 2 — Graphical depiction of N-ChIP protocol. [file Image2.jpeg]

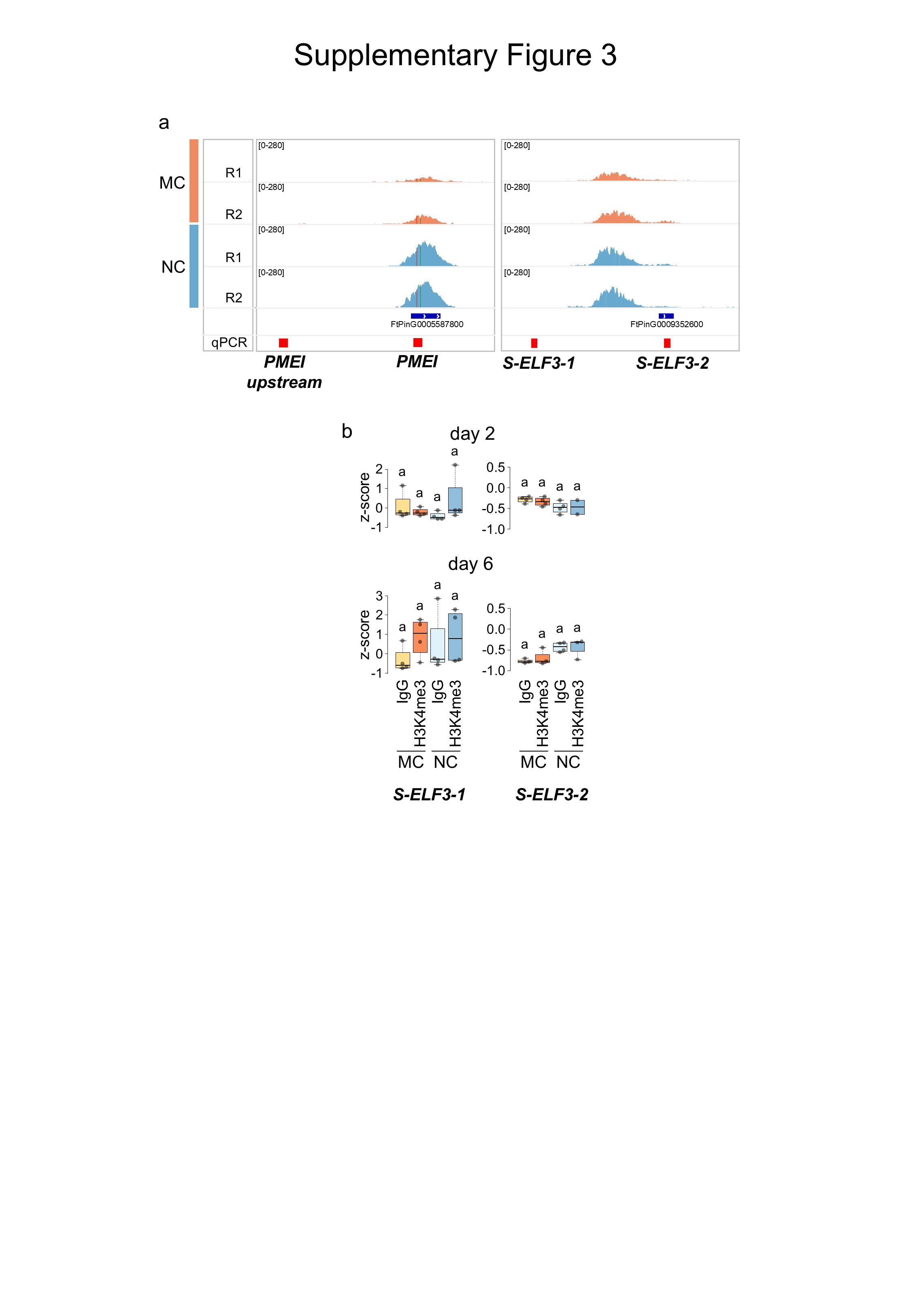

Supplement: Supplementary Figure 3 — H3K4me3 enrichment in negative control regions. (A) Visual representation of the H3K4me3 enrichment on negative control selected regions: PME upstream (Figure 4) and S-ELF3. Coverage of the replicas are represented in the same scale for each gene. Regions amplified by qPCR are indicated as red square on the bottom. (B) ChIP-qPCR of H3K4me3 levels of the negative regions around the S-ELF3 gene. Box plots show the distribution of normalised z-scores of H3K4me3%Input for MC and NC across the time points (day two and day six). [file Image3.jpeg]

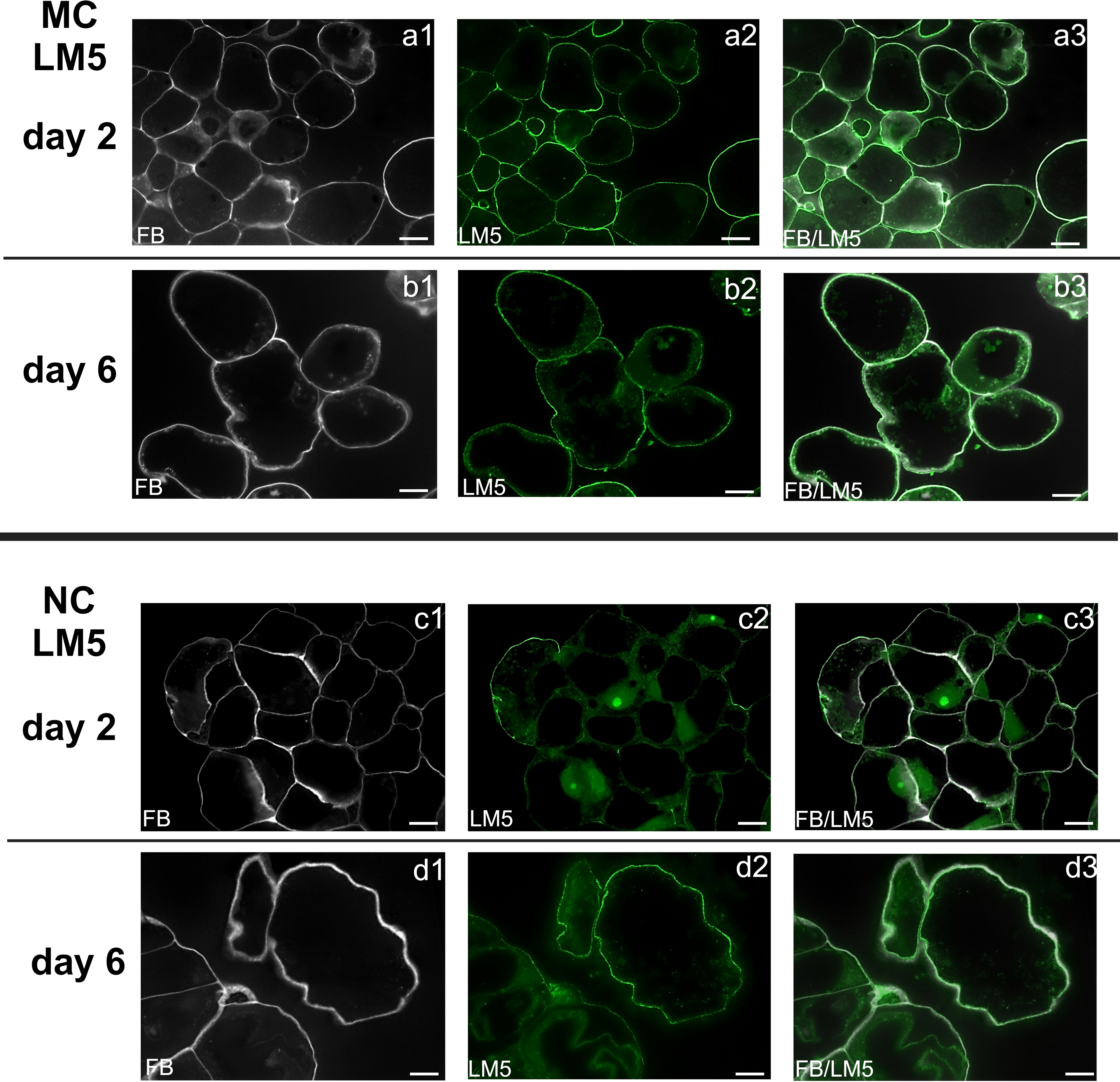

Supplement: Supplementary Figure 4 — Immunocalisation of LM5 in F. tataricum MC and NC in passage dynamics, i.e., day two and day six. FB fluorescent brightener. Scale bar: 10 μm. [file Image4.jpeg]

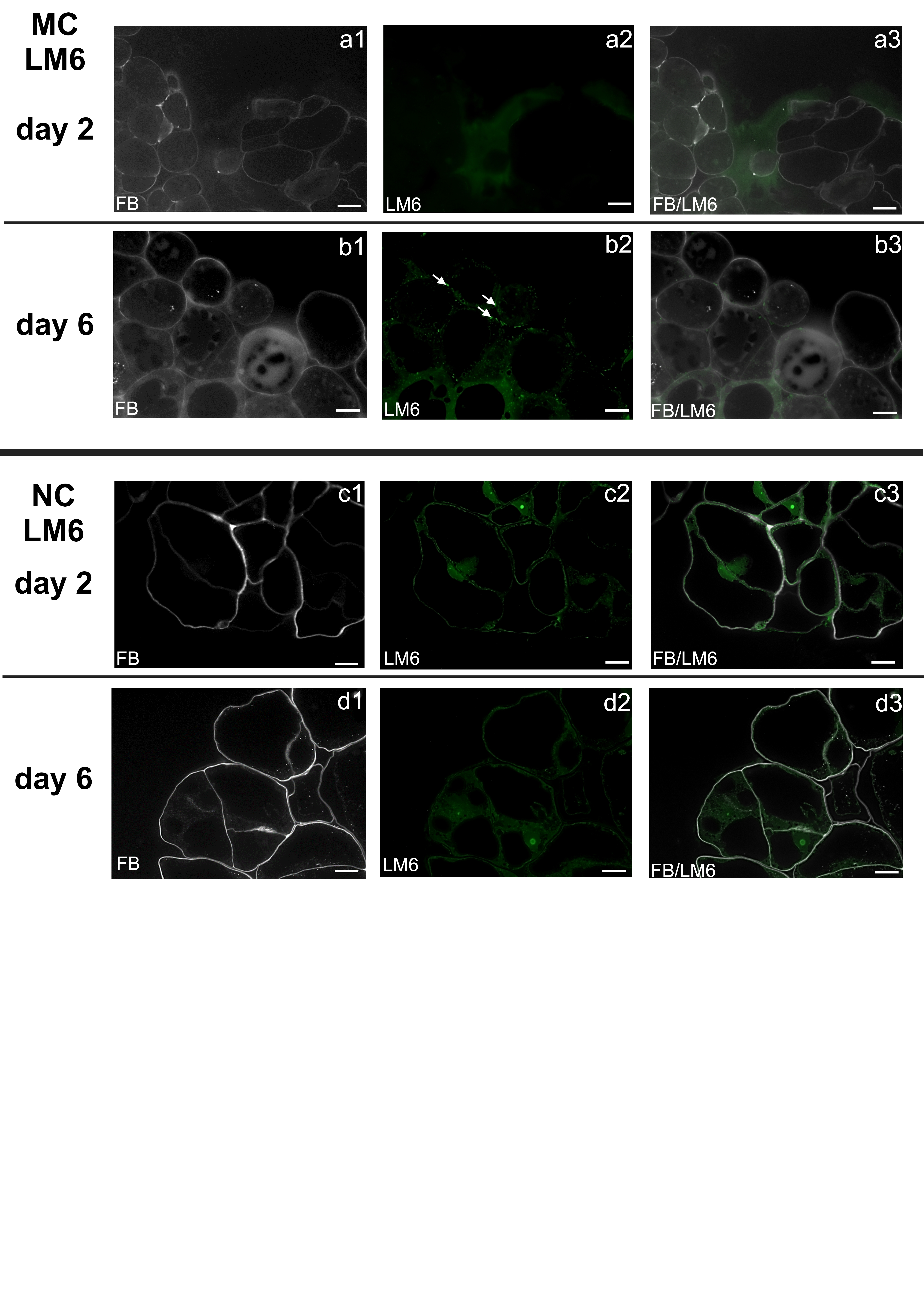

Supplement: Supplementary Figure 5 — Immunocalisation of LM6 in F. tataricum MC and NC in passage dynamics, i.e., day two and day six; white arrows- signal in the inner cell compartments. FB fluorescent brightener. Scale bar: 10 μm. [file Image5.jpeg]

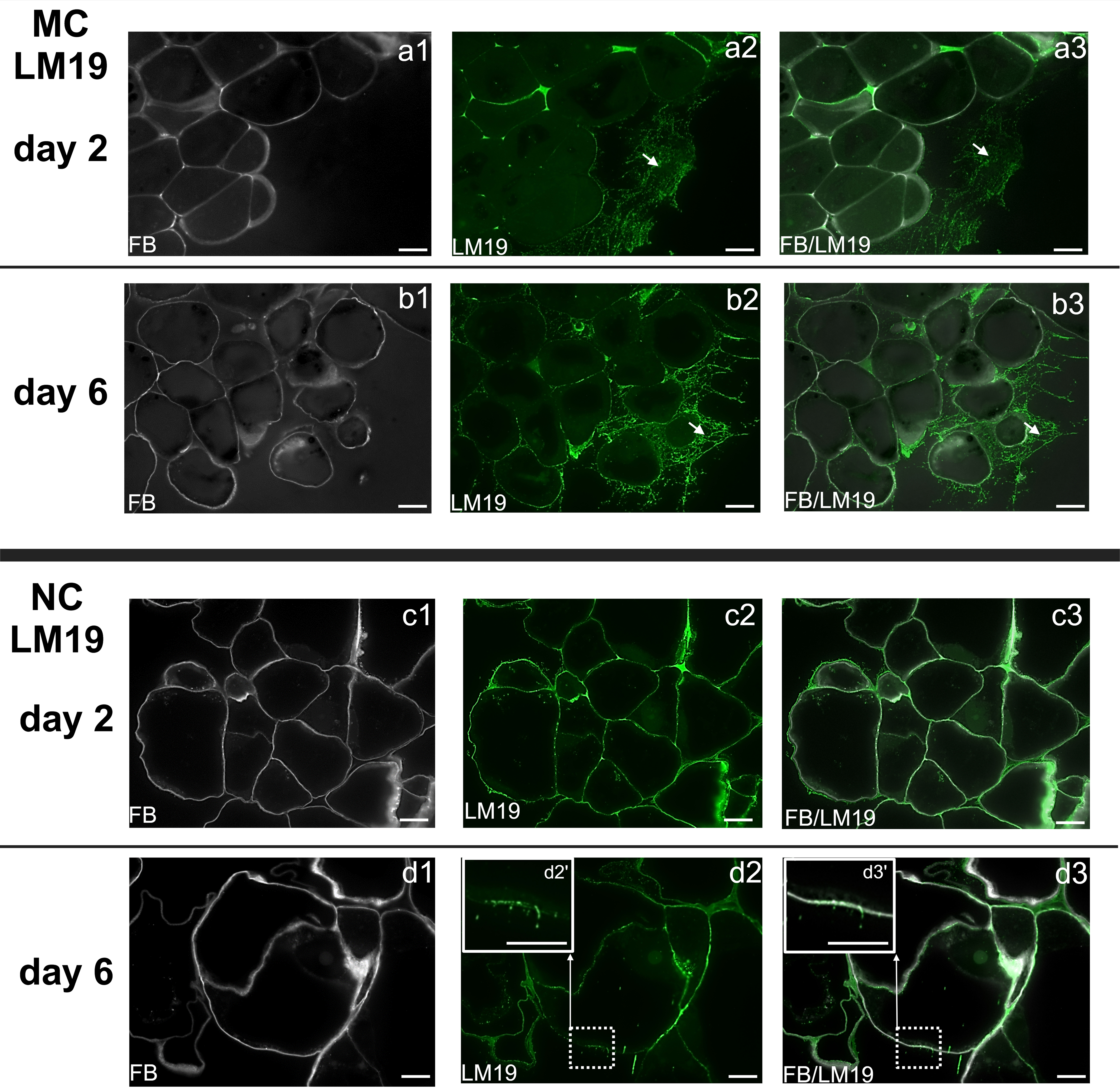

Supplement: Supplementary Figure 6 — Immunocalisation of LM19 in F. tataricum MC and NC in passage dynamics, i.e., day two and day six; signal on the callus surface:d2 and d2’ inset; d3 and d3’ inset. FB fluorescent brightener. Scale bar: 10 μm. [file Image6.jpeg]

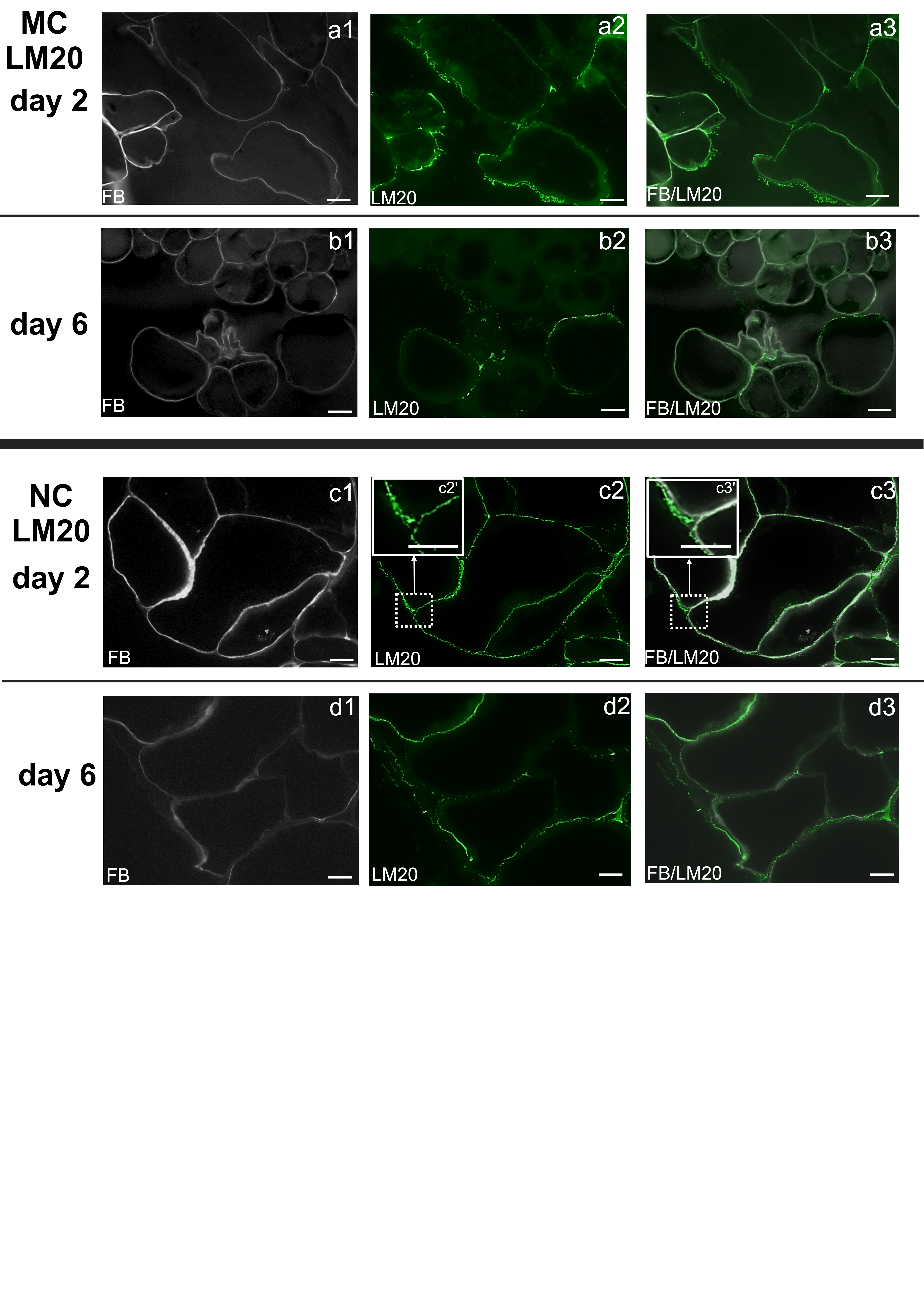

Supplement: Supplementary Figure 7 — Immunocalisation of LM20 in F. tataricum MC and NC in passage dynamics, i.e., day two and day six; signal on the callus surface: d2 and d2’ inset; d3 and d3’ inset. FB fluorescent brightener. Scale bar: 10 μm. [file Image7.jpeg]

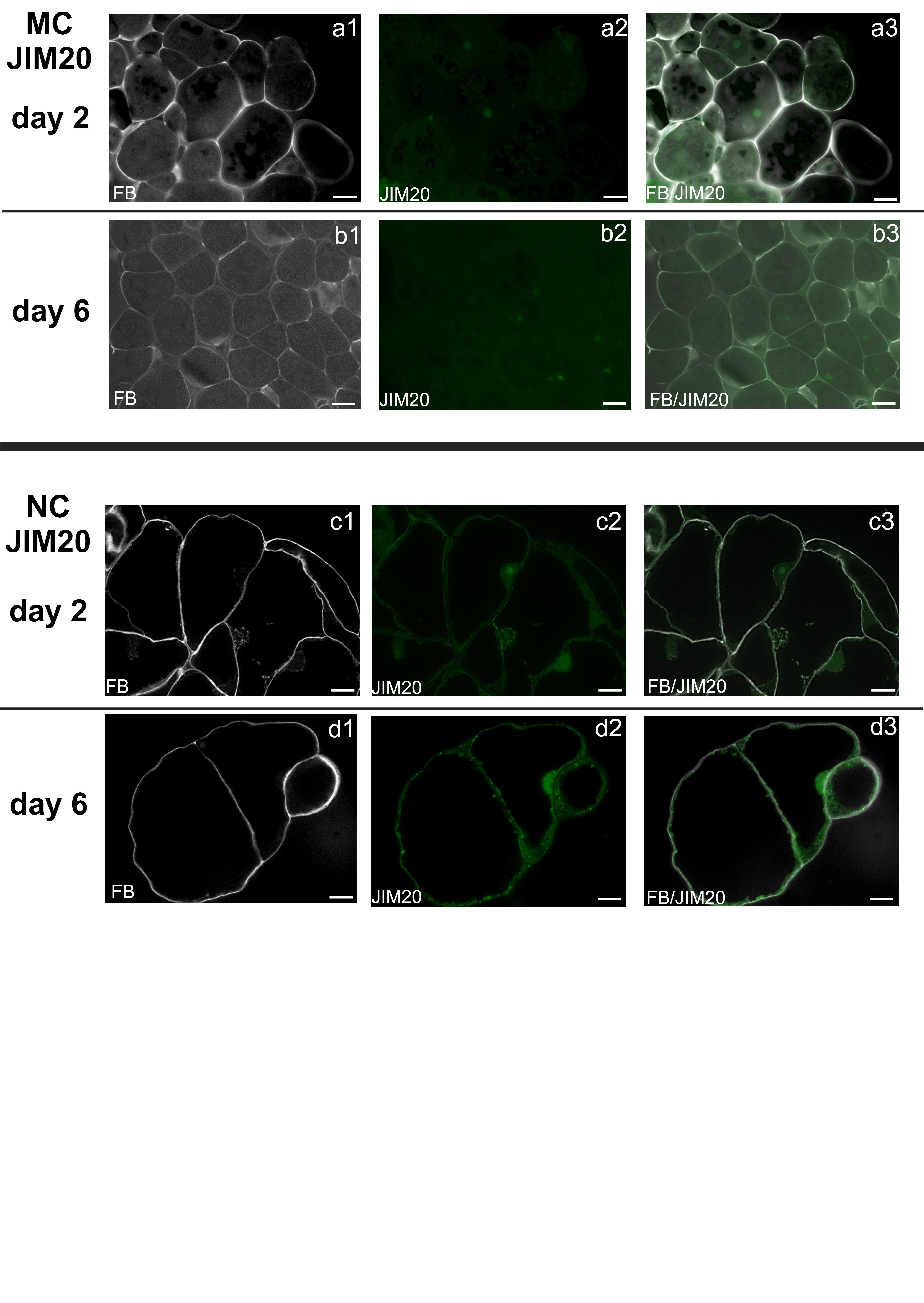

Supplement: Supplementary Figure 8 — Immunocalisation of JIM20 in F. tataricum MC and NC in passage dynamics, i.e., day two and day six. FB fluorescent brightener. Scale bar: 10 μm. [file Image8.jpeg]
