# Supplementary material for: H3K4me3 changes occur in cell wall genes during the development of Fagopyrum tataricum morphogenic and non-morphogenic calli
Source: Front Plant Sci. 2024 Sep 25;15:1465514. doi: 10.3389/fpls.2024.1465514 (PMC11461221; doi:10.3389/fpls.2024.1465514)
Supplement: Supplementary file 10 [file Table1.docx]

**Supplementary table 1. ChIP-seq alignment statistics.**

| Sample name | Target | Total reads | Mapped reads | Mapping efficiency | Unique reads | Unique ratio | Uniquely mapped reads | Uniquely mapped ratio |
| --- | --- | --- | --- | --- | --- | --- | --- | --- |
| MC_H3K4me3_1ug_rep1 | H3K4me3 | 91,456,380 | 87,976,375 | 96.19 | 55,495,641 | 63.08 | 47,059,265 | 53.49 |
| MC_input_rep1 | Input | 108,131,590 | 106,537,494 | 98.53 | 73,216,630 | 68.72 | 50,869,760 | 47.75 |
| MC_H3K4me3_1ug_rep2 | H3K4me3 | 101,548,328 | 99,505,982 | 97.99 | 63,673,837 | 63.99 | 56,311,869 | 56.59 |
| MC_input_rep2 | Input | 94,981,746 | 93,785,142 | 98.74 | 69,841,723 | 74.47 | 49,682,873 | 52.98 |
| NC_H3K4me3_1ug_rep1 | H3K4me3 | 91,972,966 | 90,489,362 | 98.39 | 60,312,429 | 66.65 | 55,903,305 | 61.78 |
| NC_input_rep1 | Input | 131,304,654 | 129,834,124 | 98.88 | 90,584,405 | 69.77 | 68,116,760 | 52.46 |
| NC_H3K4me3_1ug_rep2 | H3K4me3 | 102,511,282 | 100,931,478 | 98.46 | 70,855,865 | 70.2 | 64,021,110 | 63.43 |
| NC_input_rep2 | Input | 119,011,120 | 117,690,032 | 98.89 | 83,652,665 | 71.08 | 63,264,603 | 53.76 |

**Sample name**: MC, morphogenic callus, NC, non-morphogenic callus; H3K3me3, immunoprecipitated sample using anti-H3K4me3; input, input sample; 1ug, amount of antibody used; rep1, biological replicate 1; rep2, biological replicate 2.

**Total reads**: total number of reads sequenced.

**Mapped reads**: number of reads that mapped to the reference genome. This includes both uniquely and multimapped reads.

**Mapping efficiency**: percentage of mapped reads calculated as mapped reads divided by total reads.

**Unique reads**: number of reads after removal of PCR duplicates.

**Unique reads ratio**: percentage of unique reads calculated as unique reads divided by mapped reads.

**Uniquely mapped reads**: number of reads after removal of PCR duplicates and multimapping reads.

**Uniquely mapped ratio**: percentage of uniquely mapped reads calculated as uniquely mapped reads divided by mapped reads.
