# Supplementary material for: H3K4me3 changes occur in cell wall genes during the development of Fagopyrum tataricum morphogenic and non-morphogenic calli
Source: Front Plant Sci. 2024 Sep 25;15:1465514. doi: 10.3389/fpls.2024.1465514 (PMC11461221; doi:10.3389/fpls.2024.1465514)
Supplement: Supplementary file 11 [file Table2.docx]

**Supplementary Table 2. ChIP-seq general peak calling statistics.**

| Sample name | Target | Peaks | Avg peak width | Avg Peak Score | RiP | FRiP |
| --- | --- | --- | --- | --- | --- | --- |
| MC_H3K4me3_1ug_rep1 | H3K4me3 | 26,136 | 1723.98 | 1.95E-06 | 31950471 | 0.68 |
| MC_H3K4me3_1ug_rep2 | H3K4me3 | 27,037 | 1745.4 | 1.25E-06 | 42782113 | 0.76 |
| NC_H3K4me3_1ug_rep1 | H3K4me3 | 26,897 | 1513.62 | 6.89E-06 | 46900047 | 0.84 |
| NC_H3K4me3_1ug_rep2 | H3K4me3 | 26,837 | 1480.39 | 6.41E-06 | 49064942 | 0.77 |

**Sample name**: MC, morphogenic callus, NC, non-morphogenic callus; H3K3me3, immunoprecipitated sample using anti-H3K4me3; input, input sample; 1ug, amount of antibody used; rep1, biological replicate 1; rep2, biological replicate 2.

**Peaks**: number of peaks detected for each sample.

**Avg Peak Width**: average width of the detected peaks

**Avg Peak Score**: average score of the detected peaks.

**RiP**: Read pairs in peaks - is the number of total reads falling inside the regions detected as peaks.

**FRiP**: Fraction of reads in peaks - is the proportion of RiP over the number of mapped reads.
