# Supplementary material for: H3K4me3 changes occur in cell wall genes during the development of Fagopyrum tataricum morphogenic and non-morphogenic calli
Source: Front Plant Sci. 2024 Sep 25;15:1465514. doi: 10.3389/fpls.2024.1465514 (PMC11461221; doi:10.3389/fpls.2024.1465514)
Supplement: Supplementary file 13 [file Table4.docx]

**Supplementary Table 4. List of primers used for ChIP-qPCR experiments**

| **Gene ID** | **Name** | **Forward primer (5’-3)** | **Reverse primer (5’-3)** | | **Product size** |
| --- | --- | --- | --- | --- | --- |
| ***FtPinG0005587800*** | ***PMEI*** | TCCTGGCAAAATCAACGGCT | GGTAGGTGGTTTTGGTGCATG | | 90 |
| ***FtPinG0007594600*** | ***PME*** | GTCGCATTAGGGTTTGTCGC | ATGGAAATCAAGCCGTCGGT | | 69 |
| ***FtPinG0003136500*** | ***PX* for**  **ChIP-qPCR** | CAGGGATGTGATGCTTCGGT | | GGTGTCAATGACGTCGAATCC | 108 |
|  | ***PX* for**  **RT-qPCR** | TAGGGCAAGCAAGGTGTACG | | CTGAGGTCGGACAATTGCCT | 102 |
| ***FtPinG0002292300*** | ***EXTENSIN1*** | GGAGTCGATGCCGGAGTATG | TTAAACCGCCAACTCCACCA | | 98 |
| ***FtPinG0000702100*** | ***EXTENSIN2*** | GAGGCTTCACTGGCGGATAG | TTCCCCACCAGTGAAACCAC | | 92 |
| ***FtPinG0002932100*** | ***POLYGALACTURONASE1*** | TGGTTGCACGGACGTTGATA | ACTGTGATGTTGGACACGCA | | 111 |
| ***FtPinG0000462300*** | ***POLYGALACTURONASE2*** | CCATTGGAGGCACAAAAGGC | GGGTCGGAGCTTTTGGTGTT | | 119 |
| ***FtPinG0009352600*** | ***S-ELF3-1*** | TAAGTCGGAAGCCCACAACC | TGGACTGTTGTGGGCTGTTT | | 179 |
| ***FtPinG0009352600*** | ***S-ELF3-2*** | CCCACAACCTGCTCACAAGA | TGGGCTGTTTCATGTGTGGA | | 158 |
| ***FtPinG0009470600*** | ***PMEI upstream region*** | AGTTATTACGCGACACCGTC | TAACTACGAGACGGTGTCGC | | 29 |
