# Supplementary material for: H3K4me3 changes occur in cell wall genes during the development of Fagopyrum tataricum morphogenic and non-morphogenic calli
Source: Front Plant Sci. 2024 Sep 25;15:1465514. doi: 10.3389/fpls.2024.1465514 (PMC11461221; doi:10.3389/fpls.2024.1465514)
Supplement: Supplementary file 16 [file Table7.docx]

**Supplementary Table 7. List of antibodies used for immunohistochemistry**

| **Antibody** | **Epitope** | **References** |
| --- | --- | --- |
| **LM5** | linear tetrasaccharide in (1–4)-β-D-galactans (RG I side chain) | (Smallwood et al., 1994) |
| **LM6** | 1,5-alpha-L-arabinan | (Willats et al., 1999) |
| **LM19** | Homogalacturonan (HG) domain in pectic polysaccharides, which recognises a range of HGs with preferential binding to unesterified HGs | (Verhertbruggen et al., 2009) |
| **LM20** | HG domain in pectic polysaccharides, which requires methyl esters for recognition of HG and does not bind to unesterified HG | (Verhertbruggen et al., 2009) |
| **JIM20** | Extensin/ HRGP glycoprotein | (Jones, 1997) |
